# The PROFID project

**DOI:** 10.1093/eurheartj/ehaa645

**Published:** 2020-09-19

**Authors:** Nikolaos Dagres, Niels Peek, Christophe Leclercq, Gerhard Hindricks

**Affiliations:** Leipzig Heart Institute, Leipzig, Germany; Department of Electrophysiology, Heart Center Leipzig at the University of Leipzig, Leipzig, Germany; Centre for Health Informatics, School of Health Sciences, The University of Manchester, Manchester, UK; University of Rennes, CHU Rennes, INSERM, Rennes, France; Leipzig Heart Institute, Leipzig, Germany; Department of Electrophysiology, Heart Center Leipzig at the University of Leipzig, Leipzig, Germany

**A large European effort towards personalized prediction and prevention of sudden cardiac death after myocardial infarction funded by the European Union**

Sudden cardiac death (SCD) is the leading cause of death and accounts for ∼20% of all deaths. In most cases, it is a consequence of myocardial infarction. The advent of the implantable cardioverter-defibrillator (ICD) represents tremendous progress in the prevention of SCD. As shown in landmark trials, the ICD reduces mortality if implanted prophylactically in patients with a severely impaired left ventricular ejection fraction.^1,2^ This is due to the fact that a reduced left ventricular ejection fraction after myocardial infarction is a risk marker not only for total mortality but also specifically for sudden cardiac death.^3^

Therefore, current guidelines recommend routine implantation of ICD for primary prevention of SCD in patients with ejection fraction ≤35%.^4^ Although this practice has saved many lives, it has significant shortcomings:^5,6^

1. Due to a whole series of advances in the management of heart failure and coronary artery disease, the risk of SCD in heart failure has decreased significantly in the last 15 years.^7^ Thus, only a minority of these patients that receive an ICD for primary prevention of SCD will ever receive an appropriate ICD therapy over the lifetime of the device.^8^ Although in most patients the ICD will never be needed, all patients carry the considerable long-term risk of complications associated with the device.^9,10^
2. The majority of SCD cases in absolute numbers do not occur in patients with severely reduced ejection but in the low-risk patients with moderately reduced or preserved ejection fraction.^11,12^ The explanation for this seemingly paradoxical observation is the far larger number of patients that belong to the low-risk group of patients with ejection fraction >35%. The need to identify those patients that carry a high individual risk among this, in general low-risk group has been repeatedly stated, but the search for appropriate ways to accomplish this has been futile.^13^

Thus, it is clear that we need a radically new approach to risk stratification for SCD, away from the simple dichotomy approach based on the ejection fraction towards a personalized assessment of the *individual* risk. This would allow us to avoid the many unnecessary ICD implantations in patients with ejection fraction ≤35% and at the same time protect the patients with ejection fraction >35% that carry a high individual risk by targeted CD implantation. Such an approach would however require a radically new methodology.

This personalized approach is exactly the goal of the PROFID project (https://profid-project.eu/). PROFID is funded by the European Union over the Horizon 2020 programme and consists of two steps:

- The PROFID-Reduced trial in patients with ejection fraction ≤35% but a *low* predicted individual risk for SCD that will be randomized to ICD vs. no-ICD in a non-inferiority design.
- PROFID-Preserved in patients with EF>35% and a *high* predicted individual risk for SCD that will be randomized to ICD vs. no-ICD in a superiority design. The total number of patients in both trials will be ∼3920. The results of the trials will show whether this personalized approach can be successfully applied across the whole range of ejection fraction for the decision whether to implant an ICD for primary prevention of SCD or not.

In parallel, health economic analyses will assess the economic impact of this novel approach on health care systems.

In PROFID, all major stakeholders are represented: academic institutions with top expertise in SCD, patient organizations, large hospital chains, a large statutory health insurance company, policymakers, and state authorities across Europe (*Figure 1*). Importantly, the European Society of Cardiology is a key consortium partner and represented by the European Heart Rhythm Association.

**Figure 1:**
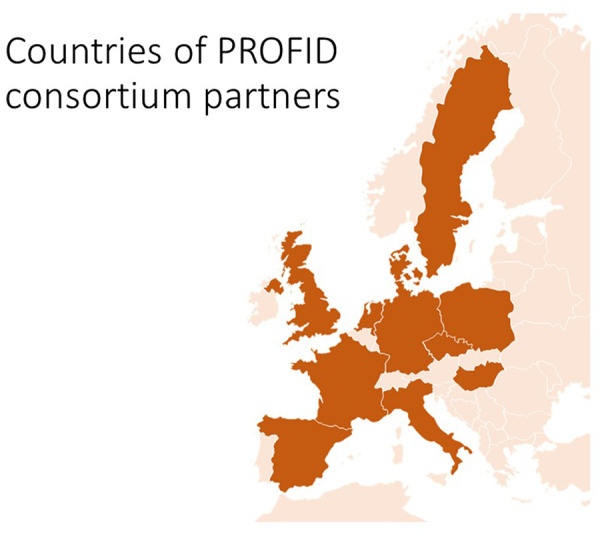
Geographic location of the PROFID consortium partners. The countries where the consortium partners are located are depicted in darker colour.

In summary, PROFID has the clear ambition to advance clinical practice and to introduce a disruptive innovation in the personalized prevention of SCD (*Figure 2*).

**Figure 2:**
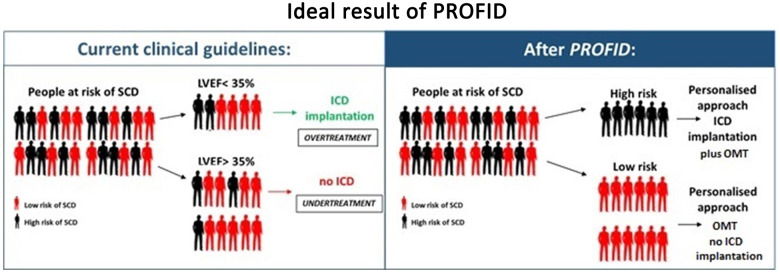
Ideal result of PROFID. On the left, the unsatisfactory current situation of imprecise identification of patients that should receive an implantable cardioverter-defibrillator based solely on the ejection fraction. On the right, the ideal result of the PROFID project: an optimal identification of high-risk patients that can be successfully protected from sudden cardiac death by implantable cardioverter-defibrillator implantation. OMT, optimal medical therapy.

## Funding


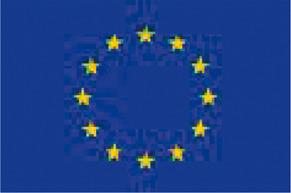


This project has received funding from the European Union's Horizon 2020 research and innovation programme under grant agreement No 847999.

**Conflict of interest:** none declared.

## References

References are available as supplementary material at *European Heart Journal* online.

## Supplementary Material

ehaa645_References_Online

