# Erratum to: The PROFID project

**DOI:** 10.1093/eurheartj/ehaa876

**Published:** 2020-11-04

**Erratum to:** The PROFID project [*Eur Heart J* 2020; doi:10.1093/eurheartj/ehaa645].

Upon the original publication of this article, the Funding section has been updated as follows online:

Previous version:

**Funding**

This project has received funding from the European Union’s Horizon 2020 research and innovation programme under grant agreement No 847999.

Corrected version:

**Funding**

**Figure:**
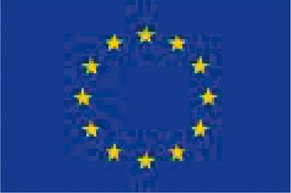


This project has received funding from the European Union's Horizon 2020 research and innovation programme under grant agreement No 847999.

The publisher apologises for this error.

