# Supplementary material for: The PROFID project
Source: Eur Heart J. 2020 Sep 19;41(39):3781–2. doi: 10.1093/eurheartj/ehaa645 (PMC7599031; doi:10.1093/eurheartj/ehaa645)
Supplement: ehaa645_References_Online [file ehaa645_references_online.docx]

**References**

1. Moss AJ, Zareba W, Hall WJ, et al. Prophylactic Implantation of a Defibrillator in Patients with Myocardial Infarction and Reduced Ejection Fraction. *N Engl J Med*. 2002;346(12):877-883. doi:10.1056/NEJMoa013474

2. Bardy GH, Lee KL, Mark DB, et al. Amiodarone or an implantable cardioverter-defibrillator for congestive heart failure. *N Engl J Med*. 2005;352(3):225-237. doi:10.1056/NEJMoa043399

3. Bauer A, Barthel P, Schneider R, et al. Improved Stratification of Autonomic Regulation for risk prediction in post-infarction patients with preserved left ventricular function (ISAR-Risk). *Eur Heart J*. 2009;30(5):576-583. doi:10.1093/eurheartj/ehn540

4. Priori SG, Blomström-Lundqvist C, Mazzanti A, et al. 2015 ESC Guidelines for the management of patients with ventricular arrhythmias and the prevention of sudden cardiac death. *Europace*. 2015;17(11):1601-1687. doi:10.1093/europace/euv319

5. Dagres N, Hindricks G. Risk stratification after myocardial infarction: is left ventricular ejection fraction enough to prevent sudden cardiac death? *Eur Heart J*. 2013;34(26):1964-1971. doi:10.1093/eurheartj/eht109

6. Dagres N, Hindricks G. Devices for management of sudden cardiac death: Successes, challenges and perspectives. *Int J Cardiol*. 2017;237:34-37. doi:10.1016/j.ijcard.2017.03.053

7. Shen L, Jhund PS, Petrie MC, et al. Declining Risk of Sudden Death in Heart Failure. *N Engl J Med*. 2017;377(1):41-51. doi:10.1056/NEJMoa1609758

8. Sabbag A, Suleiman M, Laish-Farkash A, et al. Contemporary rates of appropriate shock therapy in patients who receive implantable device therapy in a real-world setting: From the Israeli ICD Registry. *Hear Rhythm*. 2015;12(12):2426-2433. doi:10.1016/j.hrthm.2015.08.020

9. Kirkfeldt RE, Johansen JB, Nohr EA, Jorgensen OD, Nielsen JC. Complications after cardiac implantable electronic device implantations: an analysis of a complete, nationwide cohort in Denmark. *Eur Heart J*. 2014;35(18):1186-1194. doi:10.1093/eurheartj/eht511

10. Olsen T, Jørgensen OD, Nielsen JC, Thøgersen AM, Philbert BT, Johansen JB. Incidence of device-related infection in 97 750 patients: clinical data from the complete Danish device-cohort (1982–2018). *Eur Heart J*. 2019;40(23):1862-1869. doi:10.1093/eurheartj/ehz316

11. Mäkikallio TH, Barthel P, Schneider R, et al. Prediction of sudden cardiac death after acute myocardial infarction: role of Holter monitoring in the modern treatment era. *Eur Heart J*. 2005;26(8):762-769. doi:10.1093/eurheartj/ehi188

12. Myerburg RJ, Junttila MJ. Sudden Cardiac Death Caused by Coronary Heart Disease. *Circulation*. 2012;125(8):1043-1052. doi:10.1161/CIRCULATIONAHA.111.023846

13. Kutyifa V, Beck C, Brown MW, et al. Multicenter Automatic Defibrillator Implantation Trial–Subcutaneous Implantable Cardioverter Defibrillator (MADIT S-ICD): Design and clinical protocol. *Am Heart J*. 2017;189:158-166. doi:10.1016/j.ahj.2017.04.014
